# Supplementary material for: Crystal structure of the receptor binding domain of the spike glycoprotein of human betacoronavirus HKU1
Source: Nat Commun. 2017 May 23;8:15216. doi: 10.1038/ncomms15216 (PMC5529671; doi:10.1038/ncomms15216)
Supplement: Supplementary Information — Supplementary figures and supplementary tables. [file ncomms15216-s1.pdf]

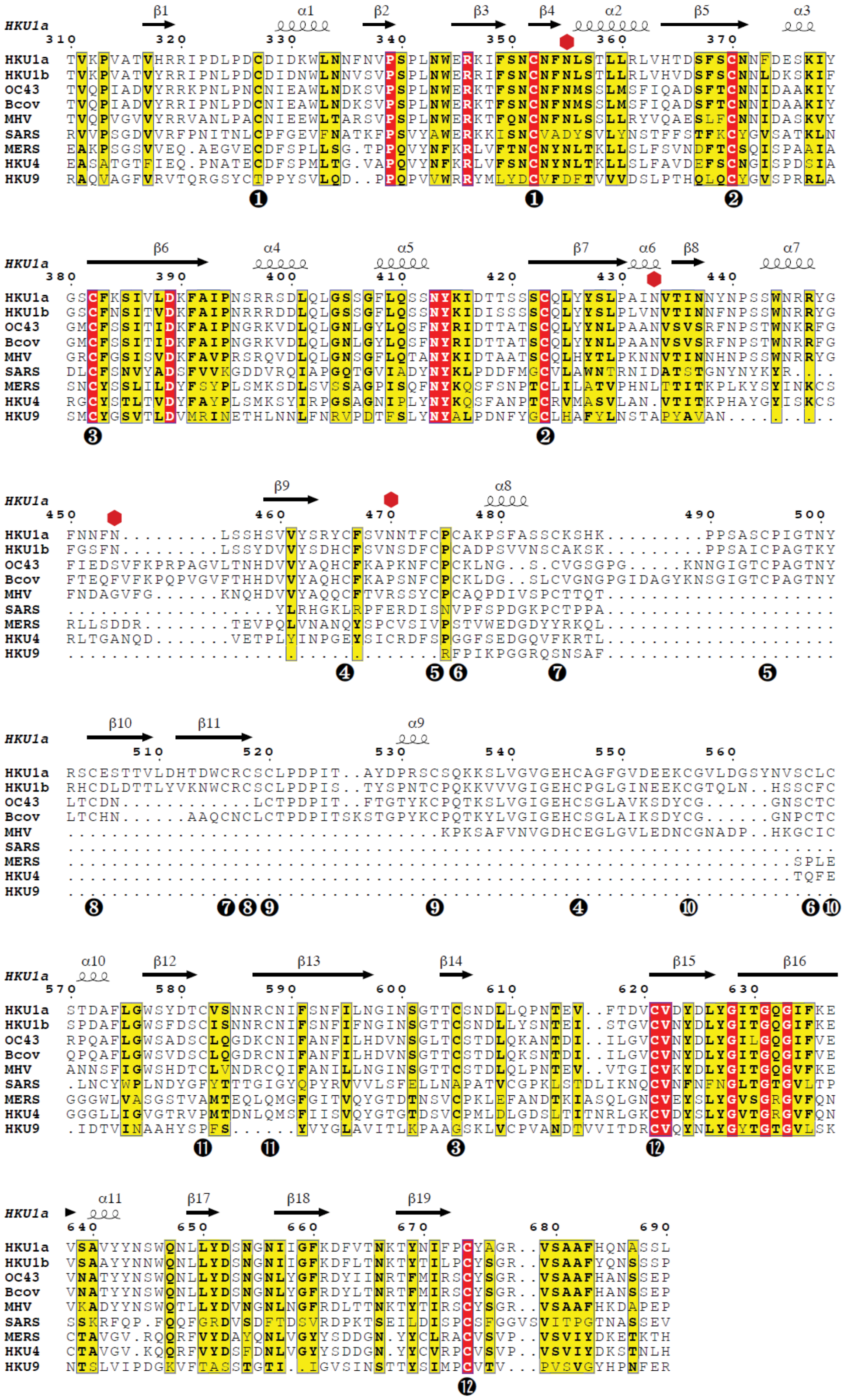

Supplementary Figure 1. Structure-based sequence alignment of CTDs of various β-CoV S proteins. HKU1 1A, HKU1 1B, OC43, BCoV, and MHV belong to lineage A β-CoV, SARS-CoV is a lineage B β-CoV, MERS-CoV and HKU4 are

lineage C β-CoVs, and HKU9 is a lineage D β-CoV. The secondary structures of HKU1A-CTD are aligned on the top of the sequences. Spiral lines indicate α-helices and arrows represent β-sheets. The number of paired Cys is shown under the alignment. Identical residues are highlighted in red and conserved residues are shown in yellow. The red hexagons represent the glycosylation sites.

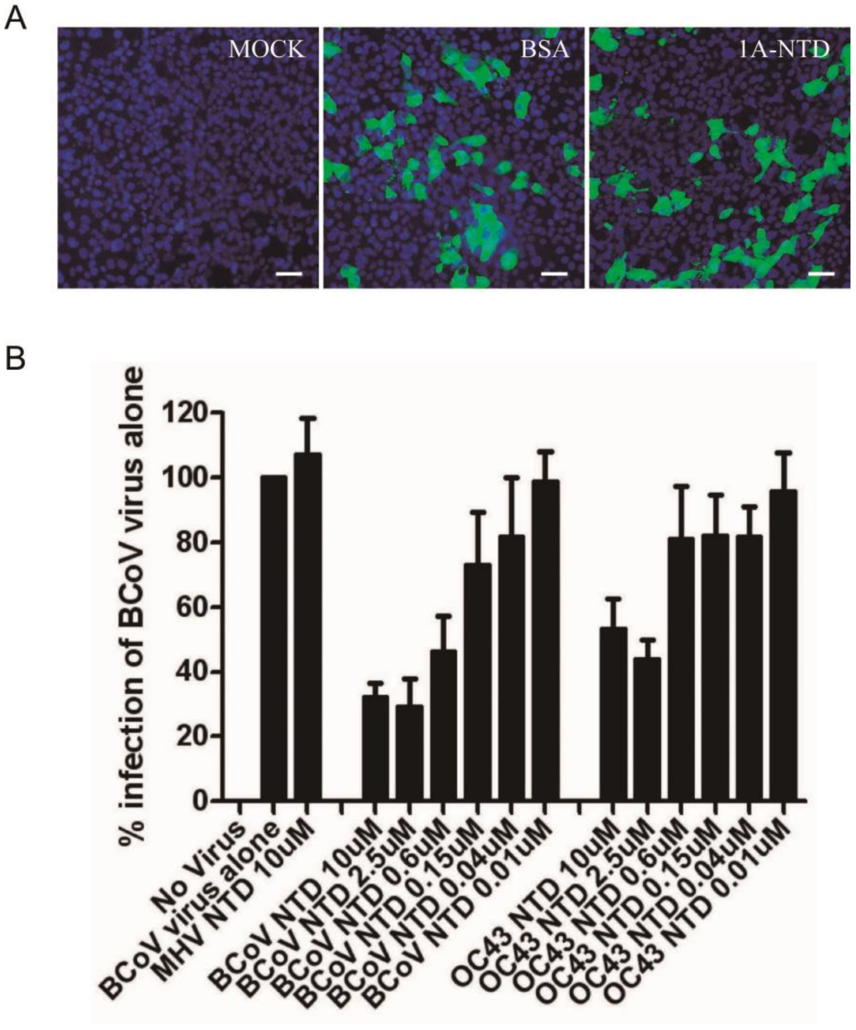

Supplementary Figure 2. Inhibition of β-CoV virus entry by NTDs. (A) No inhibition of HKU1 virus infection by HKU1 NTD. Differentiated HTBE cells were incubated with 20 μM of 1A-NTD proteins at 37ºC for 1 hr. HKU1 viruses were diluted into the same amount of proteins and added onto HTBE cells for 4 hrs. Cells were fixed and stained with polyclonal rabbit anti HKU1 S antibodies at 48 hrs post-inoculation. Scale bar = 50 μm. (B) Inhibition of BCoV virus entry by NTDs of BCoV and OC43. HRT18G cells were incubated with the indicated amount of NTD proteins for 1 hr at 37°C, and BCoV viruses were diluted into the same amount of proteins and added onto HRT18G cells. After 8hrs incubation, cells were fixed with

ice-cold methanol: acetic acid (3:1) and stained with JLantiN antibody. For each treatment, six randomized fields were selected and photographed, and the positive stained cells and total cells in each field were counted. The percentage of infection by BCoV virus alone was set as 100% and standard deviations from triplicate wells were shown as error bar.

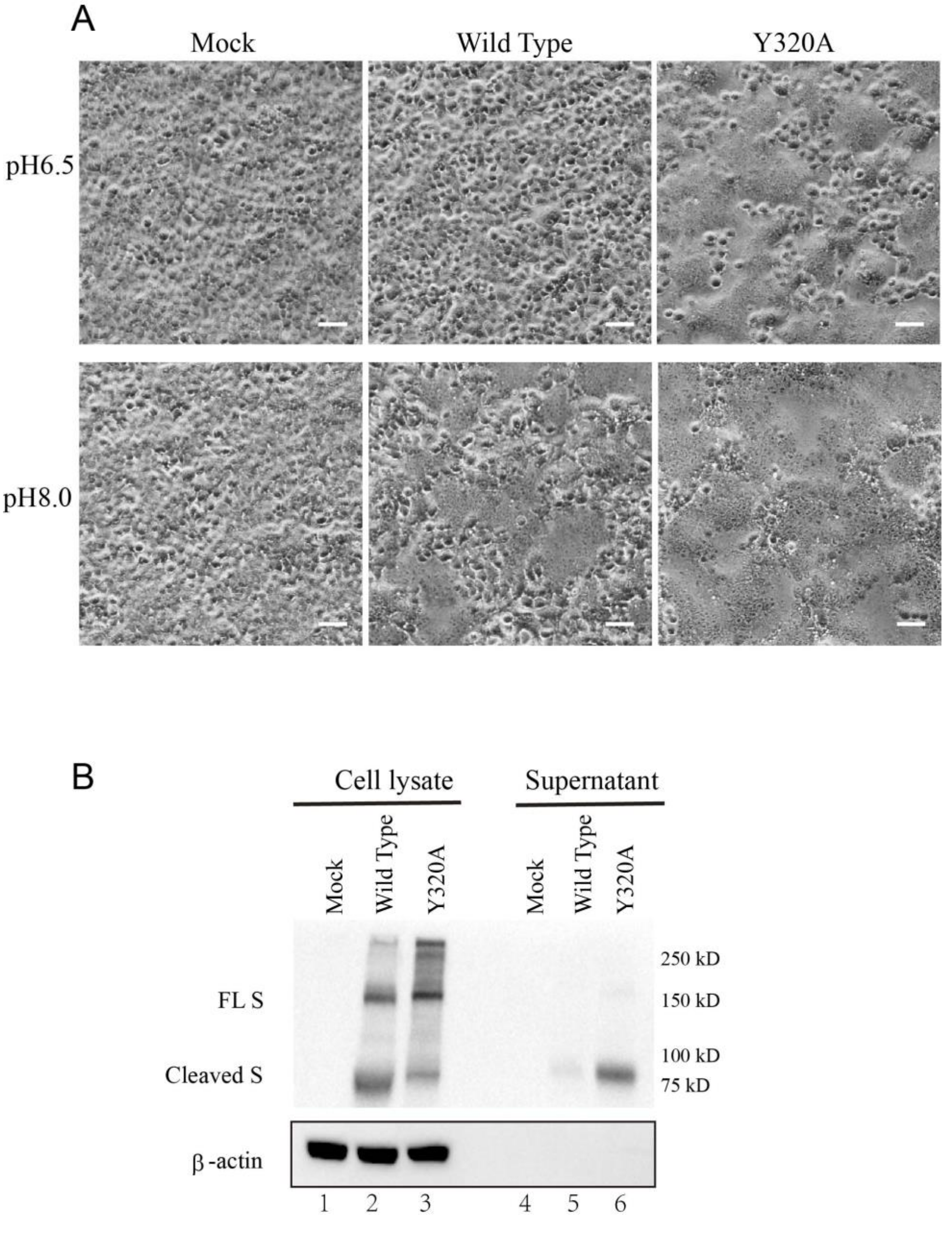

Supplementary Figure 3. Receptor- and pH- independent syncytia formation by Y320A mutant MHV S protein. (A) HEK-293T cells were transfected with plasmids encoding either wild-type or Y320A mutant MHV S protein. After 16 hrs incubation, cells were fed with the fresh medium at indicated pH. Three hours later, cells were photographed, and the representative pictures are shown. Scale bar = 50 μm. (B) Western blot. Proteins were separated on 10% SDS-PAGE and blotted with goat anti

MHV S antibody AO4 at a dilution of 1:1,000, or rabbit anti-actin antibody at a dilution of 1:2,000. Lane 1, 2, and 3 were cell lysate; Lane 4, 5, and 6 were 40-fold concentrated supernatant.

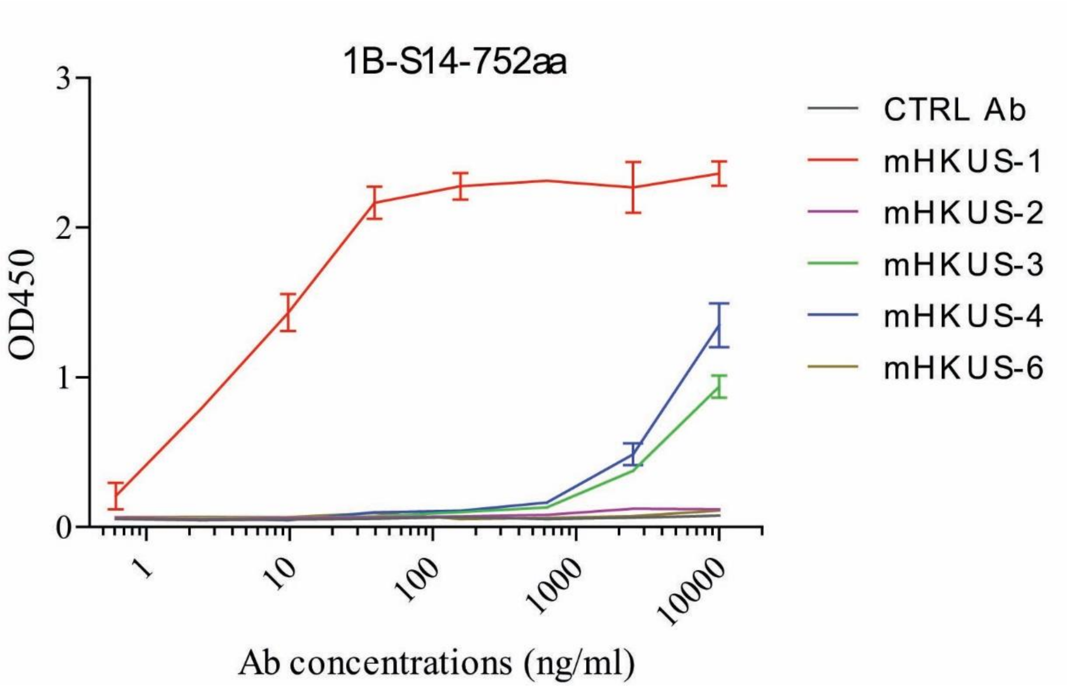

Supplementary Figure 4. Binding of mAbs to HKU1B S14-752aa. ELISA was performed using purified 1B-S14-752aa protein and purified antibodies. The experiments were done in triplicate and the standard deviations (n=3) were shown as error bar. The experiments were performed twice and one representative is shown.

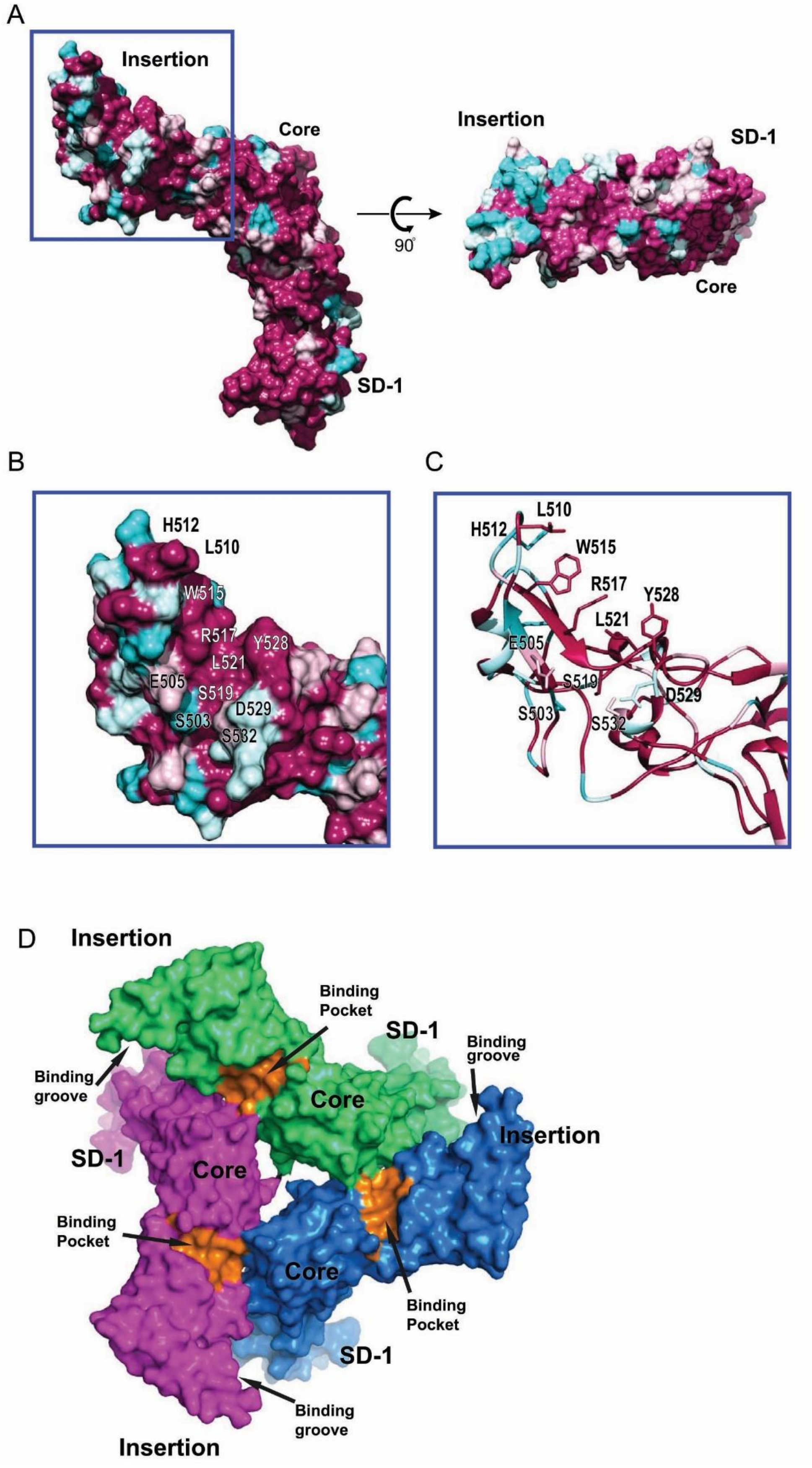
A
Insertion
Core
SD-1
90°
Insertion
SD-1
Core
B
H512
L510
W515
R517
Y528
L521
E505
S519
D529
S503
S532
C
L510
H512
W515
R517
Y528
L521
E505
S519
D529
S503
S532
D
Insertion
Binding Pocket
SD-1
Binding groove
Binding groove
Core
Insertion
SD-1
Core
Binding Pocket
Core
Binding Pocket
SD-1
Binding groove
Insertion

Supplementary Figure 5. Structure of the putative receptor binding groove and binding pocket of 1A-S310-677aa. (A) Left, side view of solvent accessible surface of HKU1 1A-S310-677aa. The orientation of 1A-S310-677aais rotated 180 degrees around the y axis with respect to 1A-S310-677aamodel in Fig 2 panel A, showing a putative receptor binding groove. The surface is rendered by conservation between sequences of HKU1 1A and HKU1 1B with a color scheme from maroon (most conserved residues) to cyan (least conserved residues). Right, top view, the orientation of the 1A-S310-677aastructure is rotated 90 degree around the x axis. (B) Magnified view of the boxed area in panel A. Residues comprising the receptor binding groove are indicated. (C) Magnified view of the boxed area in panel A shown in stick model. Residues comprising the receptor binding groove are indicated. (D) Structure of a putative binding pocket on top of the HKU1 S protein trimer. Top view of integrated HKU1S protein trimer model shown as solvent accessible surface. Three monomers are colored in blue, magenta and green, respectively. The putative binding pockets are highlighted in orange. The putative receptor binding grooves are indicated by arrows.

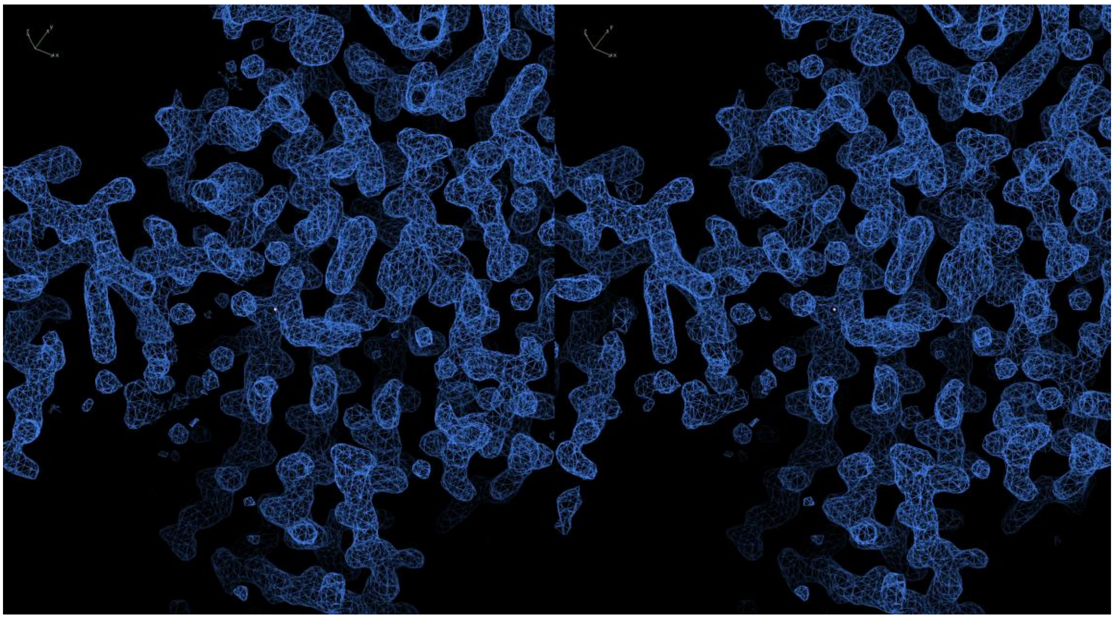

Supplementary Figure 6. Portion of the electron density map of 1A-S310-677aa crystal structure. The wall-eye stereo image of a portion of the electron density map of the crystal structure of 1A-S310-677aa (PDB ID: 5KWB) was generated using Coot v0.8.7, and the final 2Fo-Fc map is shown with blue mesh with a contour level of 1.5 σ.

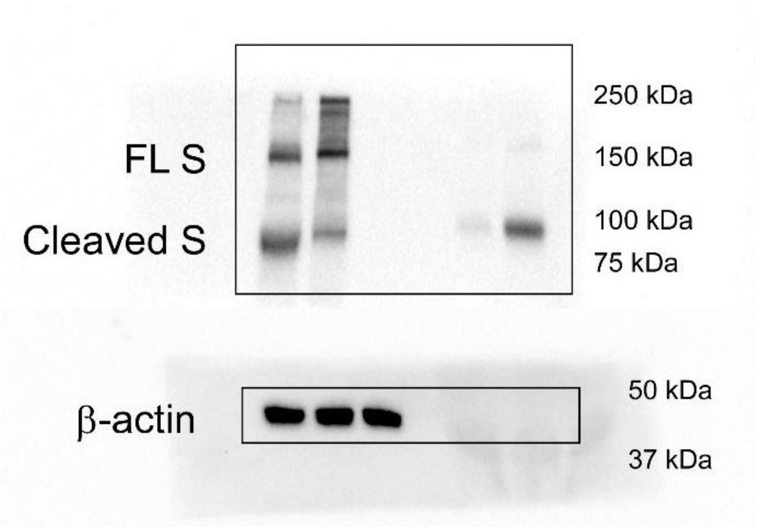

Supplementary Figure 7. Uncropped Western blot for Supplementary Figure 3B.

Supplementary Table 1. Primers used in the truncation constructs and mutagenesis

Constructs used in mutagenesis study	Forward primer sequence (5′-3′)	Reverse primer sequence (5′-3′)
14-294	ATAGGATCCGTCATAGGCGACTTCAAC	CAACTCGAGTTAGTGGTGGTGGTGGTGGTGTGTTTTGCACTGTA
14-687	ATAGGATCCGTCATAGGCGACTTCAAC	ATACTCGAGTTAGTGGTGGTGGTGGTGGTGTGCATTTTGGTGGAA
14-755	ATAGGATCCGTCATAGGCGACTTCAAC	AGTCTCGAGTTAGTGGTGGTGGTGGTGGTGACTTGAACTTGATGAT
295-755	ATAGGATCCAAATCCCTCCTTCC	AGTCTCGAGTTAGTGGTGGTGGTGGTGGTGACTTGAACTTGATGAT
310- 677	GTAGGATCCACAGTTAAGCCTGTG	ATACTCGAGTTAGTGGTGGTGGTGGTGGTGACCAGCGTAGCAT
307-687	GTAGGATCCTCAGGGTTCACA	ATACTCGAGTTAGTGGTGGTGGTGGTGGTGTGCATTTTGGTGGAA
353-687	CGCGGATCCAATTTTAATCTCTCCA	ATACTCGAGTTAGTGGTGGTGGTGGTGGTGTGCATTTTGGTGGAA
384-687	CTAGGATCCAAGAGCATTGTCCT	ATACTCGAGTTAGTGGTGGTGGTGGTGGTGTGCATTTTGGTGGAA
1B-S310-676aa	GTAGGATCCACGGTGAAACCGGTTG	ATACTCGAGTTAGTGGTGGTGGTGGTGGTGACCAGAATAGCACGGCAG

1B-S14-752aa	GTAGGATCCGTCATTGGCGACTTC	ATACTCGAGTTAGTGGTGGTGGTGGTGGTGGGAACTCGGCAGTGCGTA
1A/B-1	CTACGACGTTGTCTATTCAGATCATTGTTTCTCAGTGAACAACACATTCTG	CTCGAGACGTTGAAGGAGCCGAAGCCGTACCGCCGGTTCCAAG
1A/B-2	CTGAGCGCAATCTGTCCGGCAGGTACCAAATACCGCCACTGCGAAAGCACAACGGTG	CGGCTTCGATTTCACGCATGAATTAACCACGGACGGATCGGCACATGGGCAGAATGTGT
1A/B -3	GACCCTGTATGTGAACAATTGGTGCAGATGTTCATGTCT	GTGGTATCGACGTCGCAAGAGCGGTAGTTGGTCCC
1A/B-4	GAATACCTGCCCGCAGAAGAAATCTCTGGTCGGGG	GGGGAATACGTACTAATTGGGTCAGGCAGACATG
1A/B-5	CTCATGTTCGCCGGATGCCTTCCTGGGATGGTCC	CAACTAGAGTGATTCAGCTGGGTTCCGCACTTTTCCTCATCCAC
1A/B-6	GTGTATCAGTAACAATAGGTGCAATATTTTCAGTAAC	GAATCGAAGGACCATCCCAGGAAGGCATC
1A-V509A	GCCCTGGACCACACTGACTGGTGC	CGTTGTGCTTTCGCAAGAGCGG
1A-L510A	GCCGACCACACTGACTGGTGCAG	CACCGTTGTGCTTTCGCAAGAG
1A-D511A	GCCCACACTGACTGGTGCAGATGTT	CAGCACCGTTGTGCTTTCGC
1A-H512A	GCCACTGACTGGTGCAGATGTTCATG	GTCCAGCACCGTTGTGCTTTC
1A-T513A	GCCGACTGGTGCAGATGTTCATGTC	GTGGTCCAGCACCGTTGTG
1A-W515A	GCCTGCAGATGTTCATGTCTGCC	GTCAGTGTGGTCCAGCACCG
1A-R517A	GCCTGTTCATGTCTGCCTGACCC	GCACCAGTCAGTGTGGTCCAG
1A-L521A	CCCCTGACCCAATTACCGCCTAC	CACATGAACATCTGCACCAGTC
1A-D523A	CCCCAATTACCGCCTACGATCCC	CAGGCAGACATGAACATCTGC
1A-I525A	CCACCGCCTACGATCCCAGATC	CTGGGTCAGGCAGACATGAAC
1A-D552A	GCCGAGGAAAAGTGCGGAGTCTTG	CACGCCAAAGCCCGCGCAGTG
1A-E554A	GCCAAGTGCGGAGTCTTGGATGGG	CTCATCCACGCCAAAGCCCGC
1A-K555A	GCCTGCGGAGTCTTGGATGGGAG	TTCCTCATCCACGCCAAAGCCC
MHV-Y320A	GCCAGGAGGGTGGCAAACCTG	CACCACTCCCACTGGCTGC

Supplementary Table 2. Codon-optimized DNA sequence of HKU1 spike gene

	**Codon-optimized DNA sequence**
**HKU1 spike gene genotype A**	ATGCTGCTTATCATCTTCATTCTCCCTACTACCCTT<br>GCCGTCATAGGCGACTTCAACTGCACTAACTTTGC<br>CATTAATGACCTGAATACAACTGTGCCCCGGATAA<br>GTGAATACGTGGTGGATGTGAGCTACGGCTTGGGA<br>ACATATTATATACTCGATCGGGTGTATCTGAATAC<br>GACAATCTTGTTTACCGGGTATTTTCCTAAGAGCG<br>GAGCAAATTTTAGGGATCTGAGCCTTAAAGGGACC<br>ACCTATTTGTCAACTCTGTGGTATCAAAAGCCATT<br>CTTGTCAGATTTCAATAACGGGATCTTCAGTAGGG<br>TGAAGAACACCAAGCTGTACGTCAACAAGACCCTT<br>TACTCTGAGTTCTCCACCATCGTTATTGGCAGTGTC<br>TTTATCAATAACAGCTATACGATAGTGGTTCAACC<br>TCACAATGGTGTGTTGGAGATTACTGCGTGCCAGT<br>ACACCATGTGTGAATATCCACACACTATCTGTAAG<br>AGTAAGGGCTCTAGCCGGAATGAATCATGGCATTT<br>CGATAAATCTGAACCGCTGTGTCTGTTTAAGAAGA<br>ACTTTACATACAATGTGAGTACCGACTGGTTGTAC<br>TTCCACTTTTATCAGGAGCGGGGAACCTTCTACGC<br>TTATTACGCAGACTCCGGCATGCCAACAACATTCT<br>TGTTCTCCCTCTACCTGGGGACTCTGTTGTCACACT<br>ATTATGTCCTTCCCTTGACATGTAACGCAATAAGTT<br>CAAATACCGACAACGAAACTTTGCAGTACTGGGTG<br>ACACCCCTGTCTAAGCGCCAGTACCTTCTGAAATT<br>CGATAACAGGGGAGTCATCACCAATGCCGTGGACT<br>GTTCTAGCAGCTTCTTTAGTGAAATACAGTGCAAA<br>ACAAAATCCCTCCTTCCCAATACTGGCGTATACGA<br>TCTCTCAGGGTTCACAGTTAAGCCTGTGGCTACGG<br>TGCATAGGAGGATTCCCGATCTGCCCGACTGCGAT<br>ATCGACAAGTGGCTGAACAACTTCAACGTACCCTC<br>ACCGCTGAATTGGGAGAGGAAAATATTCTCCAATT<br>GCAATTTTAATCTCTCCACTTTGCTGAGGCTGGTCC<br>ATACCGATTCATTTAGCTGTAATAACTTTGACGAG<br>AGTAAGATATATGGCAGCTGCTTCAAGAGCATTGT<br>CCTGGACAAATTTGCCATCCCTAACTCCAGGCGCT<br>CAGATCTTCAACTGGGGAGCAGCGGATTCTTGCAG<br>AGCTCAAATTATAAAATCGATACCACAAGTAGCAG<br>TTGTCAATTGTATTACTCACTGCCCGCTATTAACGT<br>GACAATTAACAACTATAATCCTTCTTCTTGGAACC<br>GGCGGTACGGCTTCAACAACTTTAATTTGAGTAGC<br>CACTCTGTGGTGTACAGCAGATATTGTTTCTCAGT<br>GAACAACACATTCTGCCCATGTGCCAAGCCCAGCT<br>TCGCAAGTAGCTGCAAGAGCCACAAGCCGCCATCC

	GCTTCTTGTCCAATTGGGACCAACTACCGCTCTTGC<br>GAAAGCACAACGGTGCTGGACCACACTGACTGGT<br>GCAGATGTTCATGTCTGCCTGACCCAATTACCGCC<br>TACGATCCCAGATCTTGCAGCCAGAAGAAATCTCT<br>GGTCGGGGTCGGTGAACACTGCGCGGGCTTTGGCG<br>TGGATGAGGAAAAGTGCGGAGTCTTGGATGGGAG<br>TTACAATGTTAGTTGTTTGTGTAGTACTGATGCCTT<br>CCTGGGATGGTCCTATGATACTTGTGTAAGTAACA<br>ATAGGTGCAATATTTTCAGTAACTTCATCTTGAAC<br>GGAATCAACAGTGGAACCACTTGTTCCAATGACCT<br>GCTGCAACCTAATACGGAAGTGTTTACCGACGTAT<br>GCGTAGATTACGACCTTTACGGCATAACAGGCCAG<br>GGGATCTTCAAGGAAGTATCCGCGGTGTACTATAA<br>CAGCTGGCAGAACCTGCTGTACGACTCCAACGGCA<br>ACATCATTGGTTTCAAAGACTTCGTAACCAACAAG<br>ACATACAATATATTTCCATGCTACGCTGGTCGCGT<br>TAGCGCCGCATTCCACCAAAATGCATCAAGTCTGG<br>CTCTGCTGTATAGGAACTTGAAATGTTCCTACGTTC<br>TGAATAACATTAGCCTGACTACTCAGCCCTATTTT<br>GATAGCTATTTGGGATGCGTGTTTAACGCCGACAA<br>CCTGACCGACTACAGTGTATCATCCTGCGCCCTGC<br>GCATGGGGTCCGGTTTCTGCGTGGACTACAACTCA<br>CCATCATCAAGTTCAAGTCGGCGGAAGAGAAGAT<br>CAATTTCAGCTTCTTACAGATTCGTGACGTTCGAG<br>CCTTTTAATGTTTCCTTCGTCAATGACTCTATCGAG<br>AGCGTGGGCGGTCTGTACGAGATCAAGATCCCGAC<br>GAATTTCACCATTGTTGGCCAAGAGGAGTTTATTC<br>AAACCAACAGTCCTAAAGTTACGATTGACTGTTCG<br>TTGTTCGTCTGTAGTAACTACGCAGCCTGTCATGA<br>CCTTCTGAGCGAGTACGGAACCTTTTGTGATAATA<br>TCAATTCTATCCTTGATGAGGTCAATGGTCTGCTG<br>GACACAACACAATTGCACGTCGCCGACACTCTGAT<br>GCAAGGTGTGACATTAAGCTCCAATTTAAACACAA<br>ACCTTCACTTCGACGTGGACAACATTAACTTTAAG<br>TCGCTGGTGGGCTGCCTCGGTCCACACTGCGGCAG<br>CAGCTCCCGTTCATTTTTCGAGGACCTGTTGTTCGA<br>TAAAGTGAAGCTGTCAGACGTGGGATTTGTCGAGG<br>CCTACAACAATTGCACAGGAGGTTCCGAAATCAGA<br>GACCTTCTGTGCGTACAGAGCTTTAATGGCATCAA<br>GGTCTTGCCACCCATATTGTCCGAAAGTCAGATTA<br>GTGGGTATACGACTGCCGCTACAGTTGCCGCCATG<br>TTCCCTCCTTGGTCCGCCGCCGCTGGCATCCCATTT<br>TCTCTGAACGTACAGTATCGCATCAATGGCCTTGG<br>GGTGACAATGGACGTGCTGAACAAGAATCAAAAG

	CTCATCGCAACAGCTTTTAACAACGCACTGCTCTC CATACAAAACGGCTTCAGTGCGACTAATTCCGCAC TAGCGAAGATTCAGAGTGTGGTCAACTCCAATGCC CAGGCTCTCAACTCCCTTCTCCAGCAACTGTTCAA CAAGTTCGGGGCAATCAGCTCCAGTCTCCAGGAAA TTCTGTCTAGGCTGGATGCACTCGAGGCTCAGGTG CAGATAGACCGCCTGATAAATGGCCGGTTAACCGC TCTGAACGCTTATGTGAGCCAGCAGTTGTCCGACA TTAGCCTCGTTAAATTCGGCGCAGCTTTAGCAATG GAAAAAGTAAACGAGTGCGTCAAATCTCAGTCAC CCCGCATCAACTTCTGCGGGAATGGGAACCACATT CTGTCCCTAGTGCAGAATGCCCCCTACGGACTCCT GTTCATGCACTTCTCCTACAAGCCCATCTCCTTCAA AACTGTTCTCGTATCCCCTGGATTGTGCATCTCAGG GGATGTTGGAATCGCTCCAAAGCAGGGGTACTTCA TTAAGCACAACGATCACTGGATGTTCACAGGCTCA AGCTACTATTATCCAGAGCCCATTTCCGATAAGAA CGTGGTTTTCATGAACACTTGTAGCGTGAATTTCA CCAAAGCCCCACTTGTGTATCTGAATCATAGCGTG CCCAAACTGTCAGATTTCGAATCAGAACTGAGCCA CTGGTTTAAGAACCAGACTAGCATTGCCCCCAACC TTACCCTGAACCTGCATACCATTAATGCCACATTTT TAGATCTGTACTATGAAATGAATCTCATTCAGGAA TCCATTAAGTCTCTCAACAATAGCTATATAAACCT CAAAGATATAGGAACCTATGAGATGTACGTTAAAT GGCCTTGGTATGTGTGGCTACTTATCTCTTTTAGTT TCATCATCTTTCTGGTACTATTGTTTTTTATCTGTTG CTGCACTGGATGTGGATCAGCATGCTTCTCAAAGT GCCATAACTGCTGTGACGAATATGGCGGTCACCAT GACTTCGTCATAAAAACCAGCCATGATGATTGA
**HKU1 spike gene genotype B**	ATGTTTCTGATTATCTTTATTCTGCCGACCACGCTG GCTGTCATTGGCGACTTCAACTGCACGAACTCCTT TATCAACGATTACAACAAAACCATTCCGCGCATCT CTGAAGACGTGGTTGATGTTAGTCTGGGTCTGGGC ACCTATTACGTGCTGAATCGTGTTTACCTGAACAC CACGCTGCTGTTCACGGGTTATTTTCCGAAAAGCG GCGCAAACTTCCGCGATCTGGCTCTGAAAGGTTCG AAGTTTCTGAGCACCCTGTGGTATAAGCCGCCGTT CCTGTCCGACTTTAACAATGGCATCTTTTCAAAAG TGAAGAATACGAAACTGTACGTTAACAATACCCTG TATAGCGAATTCTCTACGATTGTTATCGGTTCCGTG TTTGTTAACACGTCATACACCATTGTCGTGCAGCC GCATAATGGCATTCTGGAAATCACGGCATGTCAAT

	ATACCATGTGCGAATTTCCGCATACCGTGTGTAAG<br>AGTAAGGGCTCCATCCGCAACGAAAGCTGGCACA<br>TTGACAGCTCTGAACCGCTGTGCCTGTTCAAAAAG<br>AACTTCACGTACAACGTTTACGCCGATTGGCTGTA<br>CTTTCATTTCTATCAGGAACGTGGTGTGTTTTATGC<br>GTATTACGCCGACGTCGGCATGCCGACCACGTTTC<br>TGTTCTCGCTGTATCTGGGTACCATCCTGAGCCACT<br>ATTACGTGATGCCGCTGACGTGCAACGCGATTAGT<br>TCCACCACGGATAATGAAACGCTGGAATACTGGGT<br>TACCCCGCTGTCTCGTCGCCAGTATCTGCTGAACTT<br>CGACGAACATGGCGTCATCACCAATGCCGTGGATT<br>GTTCATCGAGCTTCCTGTCTGAAATTCAGTGCAAG<br>ACCCAAAGTTTTGCGCCGAACACGGGTGTGTACGA<br>TCTGAGCGGCTTTACGGTGAAACCGGTTGCCACCG<br>TCTATCGTCGCATCCCGAACCTGCCGGATTGTGAC<br>ATTGATAATTGGCTGAACAATGTCTCAGTGCCGTC<br>GCCGCTGAATTGGGAACGTCGCATCTTTTCCAACT<br>GCAATTTCAACCTGTCAACCCTGCTGCGCCTGGTT<br>CACGTCGACAGTTTTTCCTGTAACAATCTGGATAA<br>AAGCAAGATTTTCGGTTCTTGCTTTAACAGTATCA<br>CCGTGGATAAGTTCGCGATTCCGAATCGTCGCCGT<br>GATGACCTGCAGCTGGGTTCTAGTGGCTTTCTGCA<br>ATCCTCAAACTACAAAATTGACATCTCGAGCTCTA<br>GTTGTCAGCTGTATTACAGCCTGCCGCTGGTGAAC<br>GTTACCATCAACAACTTCAACCCGTCCTCATGGAA<br>CCGCCGTTATGGTTTTGGCTCCTTCAACGTCTCGAG<br>CTACGACGTTGTCTATTCAGATCATTGTTTTAGCGT<br>GAACTCTGACTTCTGTCCGTGCGCCGATCCGTCCG<br>TGGTTAATTCATGCGTGAAATCGAAGCCGCTGAGC<br>GCAATCTGTCCGGCAGGTACCAAATACCGCCACTG<br>CGACGTCGATACCACGACCCTGTATGTGAACAATT<br>GGTGTCGTTGCAGCTGTCTGCCGGACCCGATTAGT<br>ACGTATTCCCCGAATACCTGCCCGCAGAAAAAGGT<br>CGTGGTTGGTATCGGCGAACATTGTCCGGGTCTGG<br>GCATTAACGAAGAAAAATGCGGTACCCAGCTGAA<br>TCACTCTAGTTGCTCATGTTCGCCGGACGCGTTTCT<br>GGGCTGGTCATTCGATTCGTGTATCAGCAACAATC<br>GTTGCAACATTTTTAGCAACTTCATCTTCAACGGTA<br>TCAATTCTGGCACGACCTGTAGTAACGACCTGCTG<br>TATTCCAATACGGAAGTGTCAACCGGTGTCTGCGT<br>GAACTACGATCTGTATGGCATTACCGGTCAGGGCA<br>TCTTTAAAGAAGTTTCAGCGGCCTATTACAACAAT<br>TGGCAAAACCTGCTGTACGACAGTAACGGTAACAT<br>CATCGGCTTCAAGGATTTCCTGACCAATAAGACGT

	ACACCATTCTGCCGTGCTATTCTGGTCGCGTGAGT<br>GCAGCTTTCTATCAGAACTCCTCATCGCCGGCCCT<br>GCTGTACCGTAATCTGAAATGTTCTTACGTTCTGA<br>ACAACATCTCTTTCATCAGTCAGCCGTTTTACTTCG<br>ATAGTTATCTGGGTTGCGTCCTGAACGCAGTGAAT<br>CTGACCAGCTATTCTGTGAGCTCTTGTGACCTGCG<br>CATGGGCGGTGGCTTCTGCATCGATTACGCACTGC<br>CGAGTTCCCGCCGTAAACGCCGTGGCATTTCATCG<br>CCGTATCGTTTTGTTACCTTCGAACCGTTTAATGTG<br>TCGTTTGTTAACGATAGCGTCGAAACGGTGGGTGG<br>CCTGTTTGAAATTCAGATCCCGACGAACTTCACCA<br>TCGCAGGCCATGAAGAATTTATTCAAACGAGCTCT<br>CCGAAAGTGACCATTGATTGTTCTGCTTTCGTTTGC<br>AGTAATTACGCGGCCTGTCACGACCTGCTGTCGGA<br>ATACGGTACCTTCTGCGATAACATCAACTCTATCC<br>TGAACGAAGTGAATGACCTGCTGGATATCACGCAG<br>CTGCAAGTCGCCAACGCACTGATGCAGGGCGTGAC<br>CCTGAGTTCCAATCTGAACACGAATCTGCATAGCG<br>ACGTGGATAACATCGATTTCAAGTCGCTGCTGGGT<br>TGTCTGGGCAGCCAGTGCGGTTCATCGAGCCGCAG<br>TCTGCTGGAAGACCTGCTGTTCAACAAAGTTAAGC<br>TGTCCGATGTTGGCTTTGTCGAAGCGTATAACAAT<br>TGTACCGGTGGCTCTGAAATTCGTGATCTGCTGTG<br>CGTTCAGAGTTTTAATGGTATTAAAGTCCTGCCGC<br>CGATCCTGTCCGAAACCCAAATTTCAGGTTACACG<br>ACCGCAGCTACGGTGGCAGCAATGTTCCCGCCGTG<br>GTCCGCAGCTGCGGGTGTTCCGTTTTCACTGAACG<br>TCCAGTATCGCATCAATGGTCTGGGCGTTACCATG<br>GATGTCCTGAACAAAAATCAGAAGCTGATCGCTAA<br>CGCGTTCAATAAGGCGCTGCTGAGCATTCAAAACG<br>GTTTTACGGCCACCAATTCTGCCCTGGCAAAAATT<br>CAGAGTGTCGTGAACGCTAATGCGCAAGCCCTGAA<br>CTCTCTGCTGCAGCAACTGTTCAATAAATTTGGCG<br>CAATCTCTAGTTCCCTGCAGGAAATTCTGAGCCGT<br>CTGGACAATCTGGAAGCTCAGGTTCAAATTGATCG<br>CCTGATCAACGGTCGTCTGACCGCACTGAATGCTT<br>ATGTCTCCCAGCAACTGTCAGATATTACGCTGATC<br>AAAGCAGGCGCTTCTCGCGCCATCGAAAAAGTGA<br>ACGAATGTGTTAAGAGTCAGTCCCCGCGTATCAAT<br>TTCTGCGGTAACGGCAATCATATTCTGAGCCTGGT<br>GCAAAACGCACCGTACGGTCTGCTGTTCATTCACT<br>TTTCGTATAAACCGACGAGCTTTAAGACCGTTCTG<br>GTCTCGCCGGGCCTGTGTCTGAGCGGTGATCGTGG<br>CATCGCGCCGAAGCAGGGTTACTTCATCAAGCAAA

	ACGAATCCTGGATGTTTACCGGCTCATCGTATTAC<br>TATCCGGAACCGATTAGCGATAAGAATGTTGTCTT<br>TATGAACTCATGCTCGGTGAATTTTACGAAAGCGC<br>CGTTCATCTATCTGAACAATTCTATTCCGAACCTGA<br>GTGATTTCGAAGCCGAATTTTCGCTGTGGTTCAAA<br>AATCATACCAGCATGGCACCGAACCTGACGTTCAA<br>TAGCCACATTAACGCTACCTTTCTGGATCTGTACTA<br>TGAAATGAACGTTATTCAGGAATCCATCAAGTCAC<br>TGAACAGCTCTTTCATCAACCTGAAGGAAATCGGC<br>ACCTACGAAATGTATGTGAAATGGCCGTGGTATAT<br>TTGGCTGCTGATTGTTATCCTGTTCATCATCTTTCT<br>GATGATCCTGTTTTTCATCTGCTGTTGCACCGGTTG<br>CGGCTCGGCGTGTTTTAGCAAATGCCACAATTGTT<br>GCGACGAATACGGCGGTCATAACGATTTTGTGATT<br>AAAGCCAGCCACGACGATTGA
